# Adaptive Cruise System Based on Fuzzy MPC and Machine Learning State Observer

**DOI:** 10.3390/s23125722

**Published:** 2023-06-19

**Authors:** Jianhua Guo, Yinhang Wang, Liang Chu, Chenguang Bai, Zhuoran Hou, Di Zhao

**Affiliations:** 1 College of Automotive Engineering, Jilin University, Changchun 130025, China; 2 Intelligent and Connected Vehicle Development Institute, China FAW Corporation Limited, Changchun 130013, China; 3 Key Laboratory of Bionic Engineering, Ministry of Education, Jilin University, Changchun 130025, China

**Keywords:** Adaptive Cruise Control, vehicle longitudinal dynamics, model predictive control, machine learning

## Abstract

Under the trend of vehicle intelligentization, many electrical control functions and control methods have been proposed to improve vehicle comfort and safety, among which the Adaptive Cruise Control (ACC) system is a typical example. However, the tracking performance, comfort and control robustness of the ACC system need more attention under uncertain environments and changing motion states. Therefore, this paper proposes a hierarchical control strategy, including a dynamic normal wheel load observer, a Fuzzy Model Predictive Controller and an integral-separate PID executive layer controller. Firstly, a deep learning-based dynamic normal wheel load observer is added to the perception layer of the conventional ACC system and the observer output is used as a prerequisite for brake torque allocation. Secondly, a Fuzzy Model Predictive Control (fuzzy-MPC) method is adopted in the ACC system controller design, which establishes performance indicators, including tracking performance and comfort, as objective functions, dynamically adjusts their weights and determines constraint conditions based on safety indicators to adapt to continuously changing driving scenarios. Finally, the executive controller adopts the integral-separate PID method to follow the vehicle’s longitudinal motion commands, thus improving the system’s response speed and execution accuracy. A rule-based ABS control method was also developed to further improve the driving safety of vehicles under different road conditions. The proposed strategy has been simulated and validated in different typical driving scenarios and the results show that the proposed method provides better tracking accuracy and stability than traditional techniques.

## 1. Introduction

With the continuous advancement of smart vehicles, advanced driving assistance systems (ADASs) are being utilized more frequently [1,2,3]. Among its many functions, the Adaptive Cruise Control (ACC) system stands out due to its ability to manage the vehicle’s longitudinal speed and maintain a safe distance from the vehicle in front. This feature reduces the driver’s workload, making it an integral technology in the industry [4,5].

ACC systems typically employ hierarchical control, which is composed of three layers: perception, decision and execution [6,7]. The primary role of the perception layer is to acquire the kinematic information from the vehicle and transmit it to the decision layer. There are two categories of information that this layer processes: relative motion information between the vehicle and the target vehicle (such as inter-vehicle distance and relative velocity) primarily detected via sensors including radar or camera [8]; and the vehicle’s motion state information (e.g., velocity and acceleration), which is typically obtained via onboard sensors. Nonetheless, for commercial vehicles, their high center of gravity and large load characteristics necessitate consideration of the dynamic vertical wheel load influence while designing ACC systems [9]. The most widely used method to estimate dynamic vertical wheel load involves estimating a vehicle model. However, the accuracy of the estimation using this technique is limited by the model’s precision, warranting further discussion on the credibility of the results. The decision layer determines the desired acceleration of the vehicle based on the kinematic information gathered by the perception layer and subsequently commands the execution layer to perform driving or braking operations. Finally, the execution layer includes the driving and braking system that responds to the desired acceleration issued by the decision layer, ultimately enabling the adaptive cruise function.

The operation of Adaptive Cruise Control (ACC) is influenced by various factors such as driving scenarios, engine characteristics and braking system characteristics. It can be reduced to a nonlinear optimal tracking control problem with external disturbance. In the development of the ACC system’s decision layer, several methods are applied, such as PID control, MPC control and fuzzy control. Among them, PID control is favored by the industry due to its low hardware configuration requirements and good control robustness when encountering interference. M. Canale et al. [10] used a robust PI controller to adjust the ACC system response according to a specified speed reference curve. Mahmood, Ali K et al. [11] took into consideration key action parameters of the vehicle and used two PID controllers to adjust the throttle and brake, respectively. Liang, J et al. [12] developed a vehicle acceleration controller based on parallel control theory utilizing the self-learning function of a neural network. However, the PID method requires a considerable amount of parameter adjustment work and cannot achieve optimal control in actual driving scenarios, making it an unsuitable method for ACC control. Model predictive control (MPC) can use future vehicle information for optimal multi-objective control, making it a focal point among researchers. Scholars have proposed various improvements on MPC for ACC, including Zengfu Yang’s [13] algorithm combining MPC and active disturbance rejection control, using feed forward control based on the vehicle dynamic model (VDM) and compensation control based on ADRC to improve control accuracy and suppressing the influence of internal or external disturbance. Li SB [14] uses the feedback-correction method to compensate for prediction error and improve system state prediction accuracy. The constraint-management method was utilized to soften the input/output constraints of the prediction optimization problem by modifying the cost function while avoiding calculation infeasibility caused by large tracking errors. Sangmoon Lee [15] proposed an event-triggered model predictive controller for an Adaptive Cruise Control system utilizing sampled and quantized data. Zeeshan Ali Memon [16] performed a parametric study on the MPC method to assess the response of the ACC system during critical maneuvering times. Even though the existing MPC approach is highly dependent on model accuracy, it still has great potential for improvement. Fuzzy logic has unique advantages in dealing with nonlinear problems, making it an appropriate method for designing the weight factor of the dynamic comprehensive performance index function based on fuzzy rules utilizing model predictive control [17].

Currently, two methods are primarily used for the adaptive cruise execution layer control. One method involves establishing a high-precision longitudinal dynamic model, which requires obtaining several accurate vehicle parameters for dynamic modeling [18,19]. However, changes in external conditions will affect the model’s response, making it challenging to achieve optimal performance. The other method replaces the longitudinal dynamic model with a simple linear model. Although this method is easy to implement, its anti-interference ability is poor and affects control outcomes adversely. To address this problem, this paper proposes using the integral-separated PID approach to realize actual acceleration tracking of desired acceleration [20]. This method avoids system overshoot and oscillation problems, thus enhancing the response speed and accuracy of the actuator layer.

In conclusion, this paper proposes a Fuzzy Model Predictive Control strategy for the Adaptive Cruise Control system based on a dynamic normal wheel load observer. In this control strategy, the observer provides accurate reference for brake force distribution, and the Fuzzy Model Predictive Controller ensures the ACC system’s balance between tracking performance and comfort and adapts to changing driving conditions, while the executive controller ensures the system’s response speed and execution accuracy. Finally, the joint simulation verifies the effectiveness of the observer and ACC system’s control. The main contributions of this paper are as follows:

(1) In the dynamic normal wheel load observer, Random Forest (RF) is adopted for feature recognition and dimensionality reduction and the processed data are input to the fully connected neural network (FCNN) for estimating the dynamic normal wheel load of the vehicle, which provides a new idea for estimating the vehicle’s dynamic parameters.
(2) Fuzzy rules are combined with model predictive control methods to overcome the poor adaptability of model predictive control methods to nonlinear problems. The control parameters can be adjusted according to fuzzy rules to adapt to dynamic changes, thereby enhancing the controller’s robustness.
(3) In the executive layer’s control, the integral-separate PID method is used to avoid oscillation caused by the integral action and enhance the system’s response speed, improving the system’s dynamic performance. The braking safety of the vehicle is further increased by designing a rule-based ABS control method.

The rest of the paper is organized as follows. Section 2 introduces the key component models of the vehicle, including the engine, tire, etc. Section 3 describes the specific control strategy. Section 4 discusses the simulation results, and the key conclusions of the paper are summarized in Section 5.

## 2. Vehicle and Component Model

**Figure 1:**
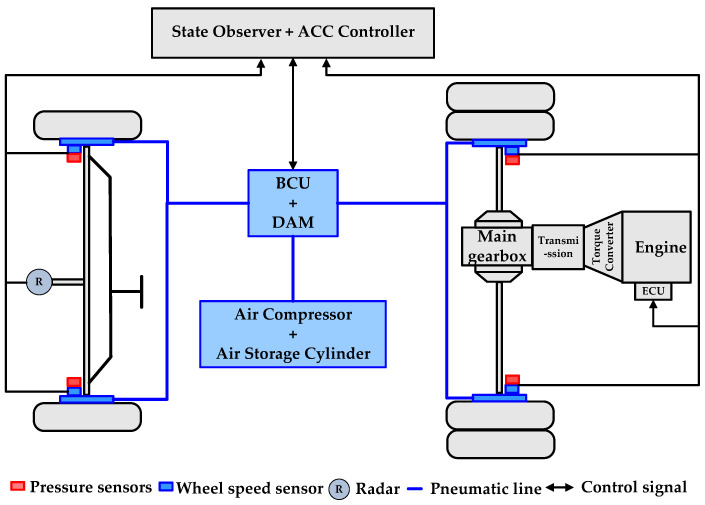
Vehicle configuration.

This paper focuses on the rear-mounted rear-drive intercity bus, as illustrated in Figure 1. The primary components of the vehicle include the radar, controller, steering system, driving system and braking system. The radar primarily obtains a target vehicle’s relative distance and velocity information. The controller consists of a state observer that detects real-time changes in dynamic vertical wheel load and an ACC system controller. The driving system incorporates the engine control unit (ECU), engine, transmission and other related parts. The braking system includes the air compressor, air storage cylinder, brake controller unit (BCU) and double channel axle modulator (DAM), the pneumatic brake actuator.

The vehicle parameters are shown in Table 1.

### 2.1. Vehicle Mathematical Model

During vehicle operation, it is necessary to balance the driving torque provided by the vehicle with various resistances, including air resistance, slope resistance and acceleration resistance. The equation expressing the driving force is presented in Equation (1).

(1) $$\frac{T_{tq}i_{0}i_{g}\eta_{t}}{r}\;=\;mg\sin\beta +\frac{1}{2}C_{D}A\rho v^{2}+mgf\cos\beta +\delta m\dot{v}$$

where *m* denotes the vehicle mass, *v* denotes the vehicle longitudinal velocity, $T_{tq}$ denotes the engine torque, $\eta_{t}$ denotes the transmission efficiency, *r* denotes the wheel rolling radius, $C_{D}$ denotes the wind resistance coefficient, *A* denotes the windward area, ρ denotes the air density, *f* denotes the road rolling resistance co-efficient, β denotes the road grade, $\delta =1+\frac{1}{m}\frac{\sum I_{w}}{r^{2}}+\frac{1}{m}\frac{I_{f}i_{g}^{2}i_{0}^{2}\eta_{t}}{r^{2}}$ denotes the rotary mass coefficient, $I_{w}$ denotes the rotating inertia of the wheels, $I_{f}$ denotes the rotating inertia of the flywheel, $i_{0}$ denotes the main reduction gear ratio and $i_{g}$ denotes the vehicle transmission ratio.

When the vehicle is in a driving state, based on the current vehicle’s required acceleration, Equation (1) can be transformed into Equation (2) to calculate the required torque of the engine under the current state.

(2) $$T_{tq}=\frac{\left(mgf\cos\beta +\frac{1}{2}C_{D}A\rho v^{2}+mg\sin\beta +\delta m\dot{v}\right)r}{i_{0}i_{g}\eta_{t}}$$

### 2.2. Engine Model

**Figure 2:**
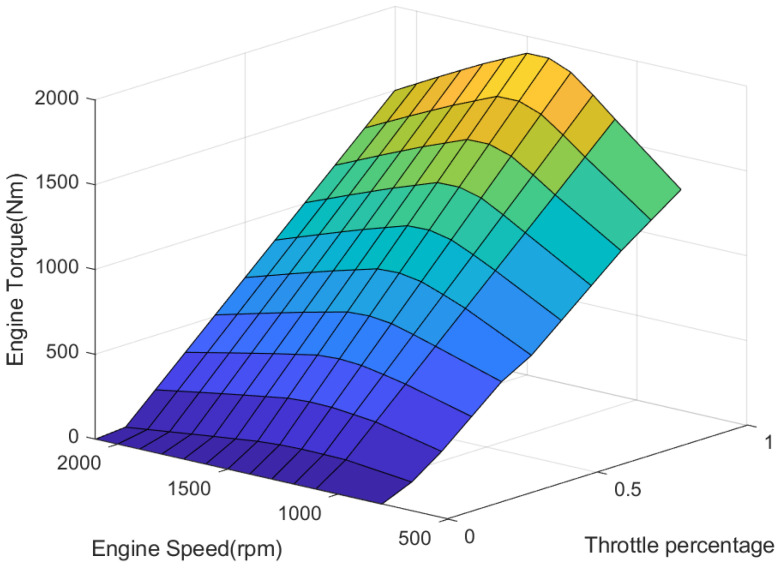
The map of the engine.

Establishing an accurate engine model is challenging due to the complexity of its physical characteristics and the numerous factors that affect its working characteristics. In this paper, based on experimental data from a diesel engine, the relationships between engine speed, torque and throttle opening were established, as Figure 2 shows.

During the driving process of the vehicle, Equation (2) can be used to obtain the corresponding driving torque value required for the expected longitudinal acceleration. Then, the value can be processed by looking up the mapping diagram of the engine torque characteristics for the ACC vehicle. By using the known engine torque $T_{tq}$ and engine speed $\omega_{e}$, the throttle opening $\alpha_{des}$ required for the expected longitudinal acceleration can be obtained, as shown in Equation (3).

(3) $$\alpha_{des}=f\left(T_{tq},\omega_{e}\right)$$

### 2.3. Brake Model

The brake system is comprised of a compressor that serves as a high-pressure air source, a storage cylinder, a double channel axle modulator (DAM) for regulating brake pressure, a brake air chamber, brake calipers and brake discs that convert pressure into brake torque. Pneumatic disc brakes offer several advantages such as a simple structure, lightweight, low noise and fast heat dissipation, which can contribute to improving vehicle active safety. The braking torque on a vehicle equipped with this brake can be computed using Equation (4) as follows:

(4) $$T_{b}=2P_{b}A_{b}\eta_{b}\mu_{b}r_{b}c_{b}.$$

where $P_{b}$ is braking pressure; $A_{b}$ is braking contact area; $\eta_{b}$ is braking efficiency; $\mu_{b}$ is braking friction coefficient; $r_{b}$ is effective braking radius; $c_{b}$ is brake coefficient. When $K_{b}=2A_{b}\eta_{b}\mu_{b}r_{b}c_{b}$, Equation (4) can be simplified as Equation (5).

(5) $$T_{b}=K_{b}P_{b}.$$

where $K_{b}$ is the brake conversion factor, Table 2 gives the brake-related parameter settings in the simulation, the units are all international system units and the brake conversion factor can be calculated as $K_{b}=0.0022$.

Corresponding to the driving state, the braking torque of the vehicle can be expressed as Equation (6).

(6) $$T_{b}=\left(\delta m\dot{v}-mgf\cos\beta -\frac{1}{2}C_{D}A\rho v^{2}-mg\sin\beta \right)r$$

Given the expected brake torque, the corresponding expected brake pressure value $P_{b}$ can be calculated according to the above formula. By comparing this value with the maximum brake pressure value of the ACC vehicle, the corresponding brake pedal opening value for vehicle deceleration control can be obtained. The calculation of the ACC vehicle’s brake pedal opening is expressed as Equation (7).

(7) $$\beta_{des}=\frac{P_{b}}{P_{b\max}}\times 100\%$$

where $\beta_{des}$ is the brake pedal opening, $P_{b}$ is the wheel demand brake pressure and $P_{b\max}$ is the upper limit of wheel brake pressure.

### 2.4. Tire Model

The tire is the sole component of a vehicle that makes contact with the ground and transmits both force and torque. Longitudinal force, lateral force and aligning torque of a vehicle arise from the interaction between the tire and the ground. Therefore, tire model accuracy is essential for creating an accurate vehicle model. Currently, tire models fall into three categories: theoretical, semi-empirical and empirical models. For this paper, the Dugoff tire model was selected to calculate tire force due to its applicability and meeting the required state estimation. The Dugoff tire model equation is shown as Equations (8)–(11).

(8) $$F_{x}=\mu F_{z}\times C_{x}\frac{\lambda }{1-\lambda }\times f\left(L\right).$$

(9) $$F_{y}=\mu F_{z}\times C_{y}\frac{\tan(\alpha )}{1-\lambda }\times f\left(L\right).$$

(10) $$f\left(L\right)=\left\{\begin{array}{c} L(2-L),L<1\\ 1,L\geq 1 \end{array}\right.$$

(11) $$L=\frac{\left(1-\lambda \right)}{2\sqrt{C^{2}\lambda^{2}+C_{z}^{2}\tan^{2}\alpha }}\times \left(1-\varepsilon \times v_{x}\times \sqrt{C_{x}^{2}\lambda^{2}+C_{y}^{2}\tan^{2}\alpha }\right).$$

where $F_{x}$ is the longitudinal force of the tire, $F_{y}$ is the lateral force of the tire, $F_{z}$ is the vertical force of the tire, $C_{x}$ and $C_{y}$ are the longitudinal slip stiffness and lateral deflection stiffness of the tire, respectively, α is the lateral slip angle of the tire, λ is the actual longitudinal slip rate and $V_{w}$ is the longitudinal velocity at the wheel center.

The slip rate represents the proportion of sliding to rolling in the vehicle’s overall motion. During braking, the rolling component of the wheel decreases while the sliding component increases as the braking intensity increases. The formula for calculating the longitudinal slip rate is presented in Equation (12).

(12) $$S_{r}=\left\{\begin{array}{ccc} \frac{r_{r0}\omega_{w}-u_{w}}{u_{w}}\times 100\% & \; & Drive\\ \frac{u_{w}-r_{r0}\omega_{w}}{u_{w}}\times 100\% & \; & Brake \end{array}\right.$$

## 3. ACC System Control Strategy Development

**Figure 3:**
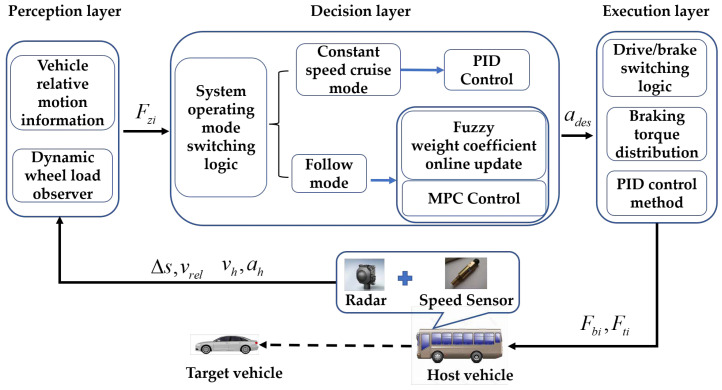
ACC control strategy architecture.

The ACC system implements a hierarchical control strategy, which consists of three main layers: perception, decision and execution. Figure 3 depicts the logical associations and data transmissions among these layers.

Vehicles equipped with ACC systems, during their driving process, obtain information from the perception layer through vehicle-mounted sensors and state observers. After a determination of the system’s working mode, the vehicle is determined to be in either constant speed cruise mode or follow mode. The PID control method is used for constant speed cruise mode. For follow mode, first the weighting factors applicable to the current scenario are obtained based on preset fuzzy rules and applied to the optimization solution of the objective function. Then, the control quantities obtained from the MPC controller are output and sent to the execution layer, finally achieving the vehicle’s Adaptive Cruise Control function.

### 3.1. Dynamic Vertical Wheel Load Observer Based on Machine Learning Methods

**Figure 4:**
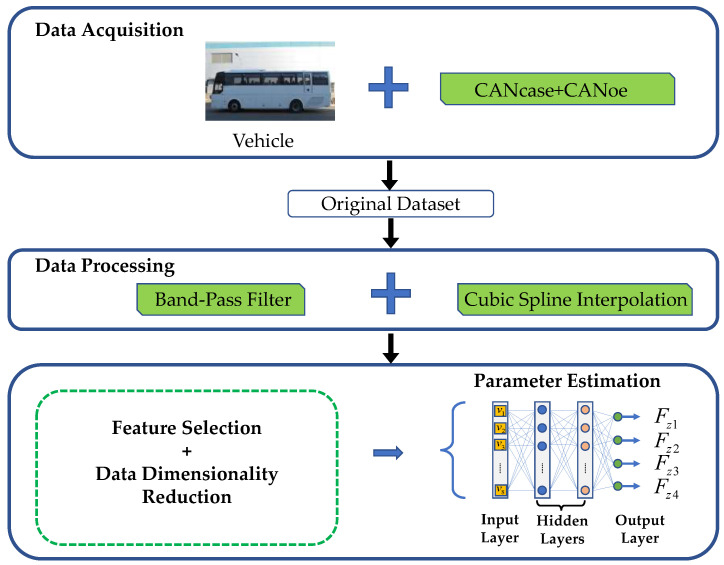
Logic structure diagram of dynamic vertical wheel load observer.

The dynamic characteristics of vehicles and the mechanical behavior of their tires exhibit strong nonlinearity. The traditional model-based frameworks typically linearize the state equations to compute tire forces, which provide accurate results when the tire is in the linear region, but they are inefficient for nonlinear conditions [21,22]. Consequently, a machine learning-driven method is proposed for estimating vehicle parameters with superior performance in tackling nonlinear problems. The framework shown in Figure 4 consists of three main stages. First, the vehicle travel data is processed and a data model is established. In the second stage, Random Forest (RF) is employed to detect essential features and reduce data dimensions because of the numerous vehicle motion state variables that may lead to high computational loads. Finally, a fully connected neural network (FCNN) is utilized to generate estimates for tire forces.

#### 3.1.1. Data Processing

The output data of vehicle sensors may contain a certain level of noise due to the variation in their sampling frequency and the vibration that occurs during driving. Therefore, it is essential to filter, resample and interpolate the data collected by the vehicle for accurate analysis. One approach to filtering anomalous data points is to use the band-pass filter technique. This method involves combining a high-pass filter and a low-pass filter either in series or parallel, resulting in a filter that permits only signals within a specific frequency range while suppressing signals beyond the cutoff frequency. The transfer function of this filter can be expressed using Equation (13).

(13) $$h\left(s\right)=\frac{Aw_{o}Bs}{s^{2}+Bs+w_{o}^{2}}.$$

where $w_{0}$ is the center frequency of the bandpass, *A* is the passband gain of the filter and *B* is the bandwidth ratio of the bandpass.

The discretized difference equation for the bandpass filter is shown in Equation (14).

(14) $$y\left(n\right)\;=\;b_{0}x\left(n\right)+\;\ldots \;+b_{M}x\left(n-M\right)-a_{1}y\left(n-1\right)-\;\ldots \;-a_{N}y\left(n-N\right).$$

where $b_{0}$–$b_{M}$ and $a_{1}$–$a_{N}$ are the system coefficients, $x_{n}$ is the input signal and $y_{n}$ is the output signal.

To ensure the continuity and smoothness of the data, the method of cubic spline interpolation is used to achieve the unification of different signals in the time and frequency domains, as shown in Equation (15).

(15) $$S\left(x\right)\;=\;a_{i}x3\;+\;b_{i}x2\;+\;c_{i}x\;+\;d_{i},\;x\;\in \;\left[x_{i},\;x_{i}+1\right].$$

where $a_{i}$, $b_{i}$, $c_{i}$, $d_{i}$ are the polynomial coefficients of the *i*-th interval, which can be obtained by solving a system of linear equations.

The coefficient matrix of the system of linear equations is a tridiagonal matrix that can be solved efficiently via the catch-up method.

#### 3.1.2. RF-Based Feature Selection and Data Dimensionality Reduction

The dataset used for neural network models often involves a considerable number of features, leading to increased complexity and difficulty in optimizing the model. To resolve this issue, the random forest technique can be implemented to remove irrelevant features from the dataset and improve the performance of the model by efficiently selecting critical features as input for the neural network.

During feature selection via the random forest algorithm, the initial dataset is randomized into training and testing sets. The random forest model is subsequently trained on the training set while recording the MDI (Mean Decrease Impurity) score for each feature. During each tree’s training process, a node within the tree randomly selects a subset of samples from its related parent node, forming out-of-bag (OOB) samples. The OOB importance value calculates the accuracy of the model’s prediction performance for each sample. Using the OOB importance scores for individual features, it becomes feasible to select substantial inputs for the neural network. The calculation of the OOB importance score for feature *j* is expressed in Equation (16).

(16) $$\mathrm{OOB}\;\mathrm{importance}j=\frac{1}{B}\sum b=1^{B}\mathrm{im}p_{j,b}.$$

where *B* represents the number of trees in the random forest, ${imp}_{j,b}$ represents the MDI value of feature *j* in the *b*-th tree, which is the information gain brought by using feature *j* to split in that tree. The OOB standard deviation value of each feature *j* can also be calculated to evaluate its stability, as shown in Equation (17).

(17) $$\mathrm{OOB}\;\mathrm{std}j=\sqrt{\frac{1}{B}\sum b=1^{B}{(\mathrm{im}p_{j,b}-\mathrm{OOB}\;\mathrm{importance}}_{j}{)}^{2}}.$$

Finally, the features are ranked based on their OOB importance values and standard deviation values and the subset of features with high importance and good stability is selected as the final feature subset.

In the RF feature screening in this paper, the results of the decision tree output were obtained using the mean method and the regression characteristics were obtained as shown in Equation (18).

(18) $$V_{Future}=\sum_{t=1}^{T}v_{b}P_{b}.$$

where $v_{b}$ is the information output by the *b*-th decision tree; $P_{b}$ is the probability distribution of the output speed information of the *b*-th decision tree.

#### 3.1.3. FCNN-Based Parameter Estimation

**Figure 5:**
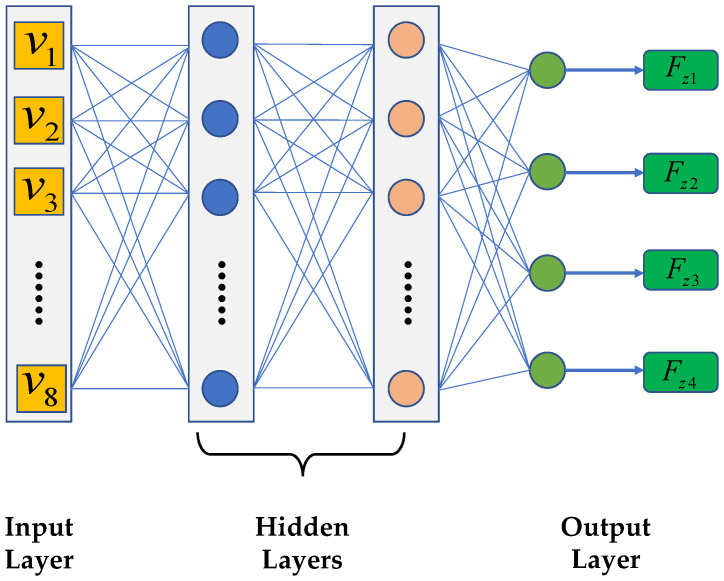
FCNN architecture.

Random forest feature selection provides the dataset for regression analysis of dynamic vertical wheel load parameters using a fully connected neural network (FCNN). The structure of the network, as displayed in Figure 5, contains input layers, hidden layers and output layers each with defined neuron count and applied activation functions. The specific hyperparameter settings are shown in Section 4.1.

The dataset is randomly partitioned into two groups consisting of a training set and a validation set. Further, to enhance training stability and rate, the training data require normalization. In this paper, Min-Max Scaling normalization, shown in Equation (19), is utilized.

(19) $$x_{norm}=\frac{x-x_{min}}{x_{max}-x_{min}}$$

where *x* is the original data, $x_{min}$ and $x_{max}$ are the minimum and maximum values in the dataset, respectively, and $x_{norm}$ is the scaled data. The range of the scaled data is usually [0,1].

The backpropagation algorithm updates the network parameters during training by using the training set data. The network’s regression performance is evaluated by utilizing the testing set after reducing the training error onto convergence. The parameter regression formula based on the fully connected neural network is demonstrated in Equation (20).

(20) $$y=f(W_{2}\cdot f\left(W_{1}\cdot x+b_{1}\right)+b_{2}).$$

In Equation (20), *x* is the input feature vector, *y* is the regression output value, $W_{1}$ and $W_{2}$ are the parameter matrices for the first and second hidden layers, respectively. $b_{1}$ and $b_{2}$ are bias vectors and *f* is the ReLU activation function. During the training process, the gradient descent algorithm is used to continuously optimize the parameters $W_{1}$, $W_{2}$, $b_{1}$ and $b_{2}$ to minimize the difference between the predicted value and the actual value.

It should be noted that this method of observing dynamic vertical wheel loads is not only applicable to highway vehicles, but also to industrial vehicles with relatively simple driving scenarios, such as forklifts. Although these vehicles have lower driving speeds, they can still exhibit changes in wheel normal loads. The observed results of dynamic vertical wheel loads can be applied to the stability issues of forklifts, such as warning of vehicle tip-over [23,24].

### 3.2. ACC Controller Decision Layer Design

The ACC system comprises a decision layer, which serves as the system’s nucleus. This layer facilitates both intelligent perception and response to complicated traffic conditions and dynamic information throughout car driving. The decision layer’s functionality includes selecting vehicle control modes and outputting expected acceleration levels to the execution layer. It derives this based on the kinematic and dynamic information received from the perception layer. Thus, it reduces the driver’s workload while increasing driving safety and comfort.

#### 3.2.1. System Operating Mode Switching Logic

**Figure 6:**
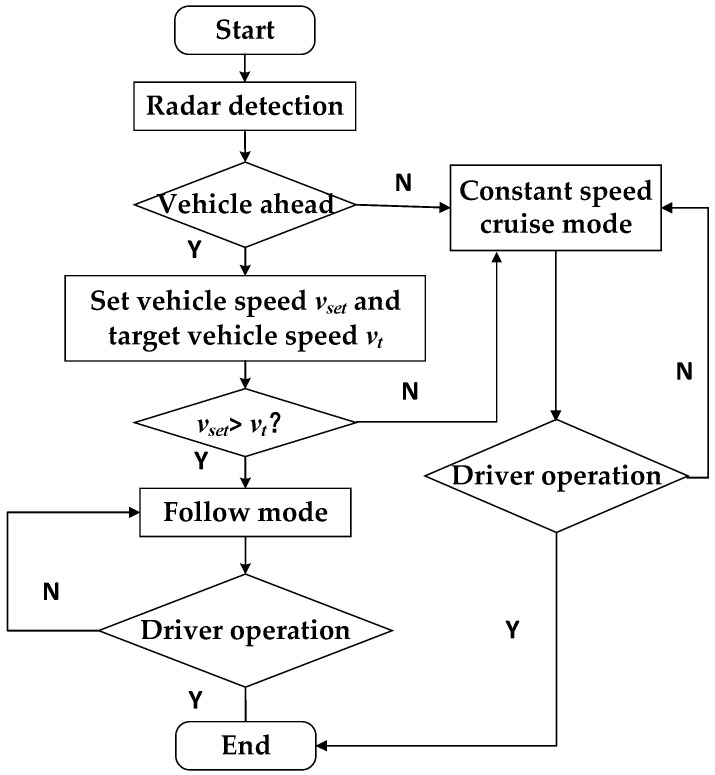
System operating mode switching flowchart.

The operation of the Adaptive Cruise Control (ACC) system involves an initial determination of the current mode of the system. If there are no vehicles detected within the radar range or if the speed of the target vehicle ahead is higher than the speed set by the host vehicle, it operates in constant speed cruise mode. However, if there is a target vehicle ahead, it switches to follow mode. A detailed illustration of this procedure is provided in Figure 6.

The Anti-Windup PID method is used to control the constant speed cruise mode. This involves using the relative speed difference and host vehicle speed as input variables and the desired longitudinal acceleration as the output variable. A saturation function is then applied to the output to limit extreme acceleration values that might otherwise affect ride stability. The primary goal of this function is to maintain vehicle stability and ensure passenger comfort.

#### 3.2.2. ACC System Follows Mode Control Strategy

The implementation of the MPC method for follow mode control necessitates the provision of the state variables needed by the controller through the perception layer. As a result of the vehicle’s inertia, there exists a time lag between the expected acceleration and the current acceleration. To represent the hysteresis properties exhibited by the two accelerations, a primary order inertial link was introduced which is described in Equation (21).

(21) $$a_{des}=\tau j(k)+a_{real}$$

where $a_{real}$ is the actual acceleration; j(k) is the acceleration change rate; $a_{des}$ is the desired acceleration; τ is the first-order inertial link time constant.

In the calculation of the desired relative spacing Δs(k), Time-To-Collision (in this paper, $t_{h}$ is used to represent TTC) is a crucial parameter [25,26,27]. The physical significance of $t_{h}$ is the time interval for possible collision calculated through the kinematic information between the vehicle and the forward obstacle, as shown in Equation (22). Combined with the current vehicle velocity $v_{h}$ and $t_{h}$, as well as the preset minimum safe distance $\Delta s_{0}$ ($\Delta s_{0}=5$ m), the desired relative spacing Δs(k) can be obtained, as shown in Equation (23).

(22) $$t_{h}=\left\{\begin{array}{cc} t_{h\_\max} & \;t_{0}-c_{v}v_{rel}>t_{h_{-}\max}\\ t_{0}-c_{v}v_{rel}-c_{a}a_{t}; & t_{h_{-}\min}<t_{0}-c_{v}v_{rel}-c_{a}a_{t}<t_{h\_\max}\\ t_{h_{-}\min} & \;\mathrm{otherwise}\; \end{array}\right.$$

(23) $$\Delta s_{des}=t_{h}v+\Delta s_{0}$$

where $t_{0}$, $c_{v}$, $c_{a}$ are constants greater than zero.

Select real-time relative spacing Δs(k), longitudinal velocity $v_{h}\left(k\right)$, relative speed $v_{rel}\left(k\right)$ ($v_{rel}=v_{t}-v_{h}$, $v_{t}$ is target vehicle velocity; $v_{h}$ is host vehicle velocity), longitudinal acceleration $a_{h}\left(k\right)$ and j(k) as the state variable. The discrete state space model of the system is established as shown in Equation (24).

(24) $$\begin{array}{c} x(k+1)=Ax(k)+Bu(k)+G\omega (k)\\ A=\left[\begin{array}{ccccc} 1 & 0 & T_{S} & -\frac{1}{2}T_{s}^{2} & 0\\ 0 & 1 & 0 & T_{S} & 0\\ 0 & 0 & 1 & -T_{S} & 0\\ 0 & 0 & 0 & 1-\frac{T_{S}}{\tau } & 0\\ 0 & 0 & 0 & -\frac{1}{\tau } & 0 \end{array}\right],B=\left[\begin{array}{c} 0\\ 0\\ 0\\ \frac{T_{S}}{\tau }\\ \frac{1}{\tau } \end{array}\right],G=\left[\begin{array}{c} \frac{1}{2}T_{s}^{2}\\ 0\\ T_{S}\\ 0\\ 0 \end{array}\right],w\left(k\right)=a_{t}\left(k\right) \end{array}$$

where $T_{s}$ is the system sampling period, $a_{t}\left(k\right)$ is target vehicle acceleration and u(k) is the expected acceleration of the ACC system at moment *k*.

Select the real-time error between the actual and the desired relative spacing δ(k), $v_{rel}\left(k\right)$, $a_{h}\left(k\right)$ and j(k) as the optimized performance target of the ACC control system. This gives $y\left(k\right)={\left[\delta \left(k\right),v_{rel}\left(k\right),a_{h}\left(k\right),j\left(k\right)\right]}^{T}$.

Thus, the expression for y(k) can be written in the form of Equation (25).

(25) $$\begin{array}{c} y(k)=Cx(k)-Z\\ C=\left[\begin{array}{ccccc} 1 & -t_{h} & 0 & 0 & 0\\ 0 & 0 & 1 & 0 & 0\\ 0 & 0 & 0 & 1 & 0\\ 0 & 0 & 0 & 0 & 1 \end{array}\right],Z=\left[\begin{array}{c} \Delta s_{0}\\ 0\\ 0\\ 0 \end{array}\right] \end{array}$$

The simplified vehicle dynamic characteristics and external interferences can cause errors between the predicted and actual values of the ACC system’s prediction model. These errors can be mitigated through feedback correction, improving the response accuracy and robustness of the system. Equation (26) demonstrates how the prediction error at a given time can be calculated using the feedback-correction principle.

(26) $$e\left(k\right)=x\left(k\right)-x_{p}\left(k/k-1\right).$$

where e(k) is the prediction error; x(k) is the actual state of the system at the moment *k*; $x_{p}\left(k/k-1\right)$ is the predicted value of moment k−1 for moment *k*.

Using a weighted matrix to adjust the prediction error to improve the system prediction accuracy, Equation (24) is transformed into Equation (27).

(27) $$x_{p}\left(k+1/k\right)=Ax\left(k\right)+Bu\left(k\right)+G\omega \left(k\right)+We\left(k\right).$$

where $W=\mathrm{diag}\left(w_{1},w_{2},w_{3},w_{4},w_{5}\right)$, $w_{i}\in \left(0,1\right)$.

Due to the safety requirements, vehicle performance and road traffic regulations in the working process of the ACC system, the real-time relative spacing Δs(k), relative velocity $v_{rel}\left(k\right)$ and other variables need to be limited within a reasonable range. The constraints are shown in Table 3.

Evaluation indicators for the ACC system include safety, following distance and comfort. Safety refers to maintaining a reasonable distance between front and rear vehicles to prevent collisions. Following distance pertains to matching the speed of the target vehicle and adjusting the actual spacing to the desired level. Comfort involves avoiding rapid acceleration and deceleration during driving. By optimizing control variables and prediction errors while accounting for various performance constraints and evaluation indicators, the ACC system can more effectively track target vehicles. The objective function is displayed in Equation (28).

(28) $$\begin{array}{cc} \; & J=\sum_{i=1}^{p}{\left[{\hat{y}}_{p}\left(k+i|k\right)-y_{r}\left(k+i\right)\right]}^{T}Q\left[{\hat{y}}_{p}\left(k+i|k\right)-y_{r}\left(k+i\right)\right]\\ \; & +\sum_{i=0}^{m-1}u{\left(k+i\right)}^{T}Ru\left(k+i\right) \end{array}$$

where ${\hat{y}}_{p}\left(k+i|k\right)$ is the performance index vector in the prediction time domain; $y_{r}\left(k+i\right)=\varphi^{i}y\left(k\right)$ is the reference trajectory; $\varphi =\mathrm{diag}\left(\rho_{\delta },\rho_{v},\rho_{a},\rho_{j}\right),\rho \in \left(0,1\right)$; *Q* is the following weight coefficient; *R* is the comfort weight coefficient; *p* is the prediction time domain; *m* is the control time domain; u(k+i) is the control variable.

From Equation (28), it can be seen that the selection of the following weight coefficient *Q* and the comfort weight coefficient *R* will directly affect the performance of the objective function. The following ability refers to the ability of a vehicle equipped with an ACC system to converge to the relative distance error and relative velocity error between itself and the target vehicle, while the comfort weight coefficient affects the control variable *u*, that is, the host vehicle acceleration. By adjusting *Q* and *R*, the system control parameter matching for coping with complicated scenarios can be achieved.

The control matrix constraints of the ACC system are organized as Equation (29).

(29) $$\begin{array}{c} \left.\begin{array}{c} \bar{M}\leq \bar{L}{\hat{X}}_{p}\left(k+p\right)\leq \bar{N}\\ U\left(k+m\right)\leq U_{\max}\\ -U\left(k+m\right)\leq -U_{\min} \end{array}\right\}\\ M=\left[\begin{array}{c} \Delta s_{0}\\ v_{\min}\\ a_{\min}\\ j_{\min} \end{array}\right],N=\left[\begin{array}{c} inf\\ v_{\max}\\ a_{\max}\\ j_{\max} \end{array}\right],L=\left[\begin{array}{ccccc} 1 & 0 & 0 & 0 & 0\\ 0 & 1 & 0 & 0 & 0\\ 0 & 0 & 0 & 1 & 0\\ 0 & 0 & 0 & 0 & 1 \end{array}\right],\bar{M}=\left[\begin{array}{c} M\\ M\\ \vdots \\ M \end{array}\right]\\ U\left(k+m\right)=\left[\begin{array}{c} u(k)\\ u(k+1)\\ \vdots \\ u(k+m-1) \end{array}\right]\bar{N}=\left[\begin{array}{c} N\\ N\\ \vdots \\ N \end{array}\right],\bar{L}=\left[\begin{array}{cccc} L & 0 & 0 & 0\\ 0 & L & 0 & 0\\ 0 & 0 & \ddots & 0\\ 0 & 0 & 0 & L \end{array}\right]\\ U_{\max}=\left[\begin{array}{c} u_{\max}\\ \vdots \\ u_{\max} \end{array}\right],U_{\min}=\left[\begin{array}{c} u_{\min}\\ \vdots \\ u_{\min} \end{array}\right] \end{array}$$

where inf stands for infinity, which means there is no upper limit to the relative spacing constraint between the ACC vehicle and the target vehicle.

Based on the above analysis, the multi-objective optimized ACC system control algorithm is turned into an online quadratic optimization problem with constraints as shown in Equation (30).

(30) $$\begin{array}{c} \left\{\begin{array}{c} \min_{U(k+m)}\left\{U{\left(k+m\right)}^{T}K_{1}U\left(k+m\right)+2K_{2}U\left(k+m\right)\right\}\\ s.t.\;\;\Omega U(k+m)\leq T \end{array}\right.\\ \Omega =\left[\begin{array}{c} \bar{L}\bar{B}\\ -\bar{L}\bar{B}\\ I\\ -I \end{array}\right],T=\left[\begin{array}{c} \bar{N}-\bar{L}\bar{G}\omega \left(k+p\right)-\bar{L}\bar{A}x\left(k\right)-\bar{L}\bar{W}e_{x}\left(k\right)\\ -\bar{M}+\bar{L}\bar{G}\omega \left(k+p\right)+\bar{L}\bar{A}x\left(k\right)+\bar{L}\bar{W}e_{x}\left(k\right)\\ U_{\max}\\ -U_{\min} \end{array}\right]\\ \bar{A}=\left[\begin{array}{c} A\\ A^{2}\\ \vdots \\ A^{p} \end{array}\right],\bar{B}=\left[\begin{array}{cccc} B & 0 & \ldots & 0\\ AB & B & \ldots & \vdots \\ \ldots & \ldots & \vdots & 0\\ A^{p-1}B & A^{p-2}B & \ldots & A^{p-m}B \end{array}\right]\\ \bar{G}=\left[\begin{array}{cccc} G & 0 & \cdots & 0\\ AG & G & \ldots & \vdots \\ \ldots & \ldots & \vdots & 0\\ A^{p-1}G & A^{p-2}G & \ldots & G \end{array}\right],\bar{H}=\left[\begin{array}{c} H_{1}\\ H_{2}\\ \vdots \\ H_{p} \end{array}\right] \end{array}$$

#### 3.2.3. Fuzzy Control Design Method for Variable Weight Coefficient Design

The effectiveness of Adaptive Cruise Control (ACC) depends on how well it can navigate the complex traffic environment encountered by a vehicle. To ensure control robustness and scenario adaptability in varying driving scenarios, variable system control parameters for ACC are required. This paper proposes the use of a weight factor for the comprehensive performance index function that is updated in real time based on the vehicle’s driving state. The weight factor comprises two components, *Q* and *R*, which correspond to the following performance index and comfort performance index, respectively. The comfort performance index weight factor was fixed as R=1. However, the online weighting coefficient parameter identification and optimization were performed by adjusting *Q*. The error between the actual and the desired relative spacing δ(k) and relative velocity $v_{rel}\left(k\right)$ were used as input variables, whereas *Q* was utilized as the output variable for fuzzy controller design. Fuzzy linguistic variables were employed to establish the weight factor *Q* of the followability index, as shown in Equation (31).

(31) $$\begin{array}{cc} \; & \delta (k):\{\mathrm{NB},\mathrm{NS},\mathrm{ZO},\mathrm{PS},\mathrm{PB}\}\\ \; & v_{rel}\left(k\right):\left\{\mathrm{NB},\mathrm{NS},\mathrm{ZO},\mathrm{PS},\mathrm{PB}\right\}\\ \; & Q:\{\mathrm{ZO},\mathrm{PS},\mathrm{PM},\mathrm{PB}\} \end{array}$$

where NB, NS, ZO, PS, PM, PB represent negative big, negative small, zero, positive small, positive medium and positive big, respectively.

**Figure 7:**
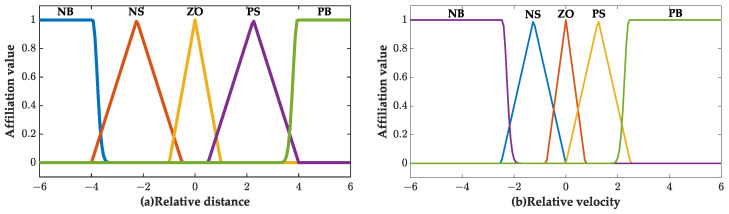
Membership function of each variable. (**a**) Error between the actual and the desired relative distance δ(k); (**b**) Relative velocity $v_{rel}\left(k\right)$.

In the fuzzy process, the fuzzification range of the real-time error between the actual and the desired relative spacing δ(k) is set as [−30,30] m and the fuzzification range of relative velocity $v_{rel}\left(k\right)$ is set as [−20,20] m/s. When Q>1, it means that the current driving condition requires a higher following index than the comfort index. Both input variables are designed by using the affiliation function of a triangle. The image of the function is shown in Figure 7.

**Figure 8:**
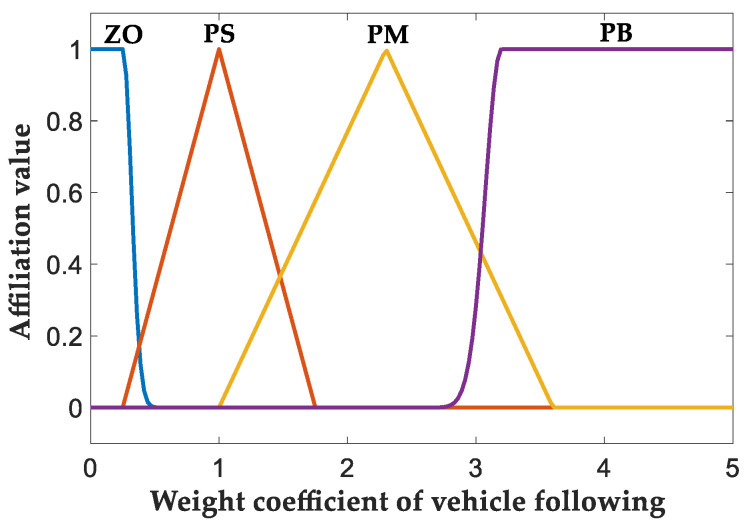
Output variable affiliation function.

The change range of *Q* is set to [0,5] and through analysis it is considered that if the value of *Q* is at [0,1], the driving state at that moment is judged to be based on the optimization goal of drive comfort, and if the value is at [1,5], the driving state at that moment is judged to be based on the optimization goal of vehicle following, so this paper is set in this part to be able to quickly respond to the desired optimization value of the Gaussian-type affiliation function and its affiliation. The function image is shown in Figure 8.

The fuzzy rules designed in this paper are shown in Table 4.

**Figure 9:**
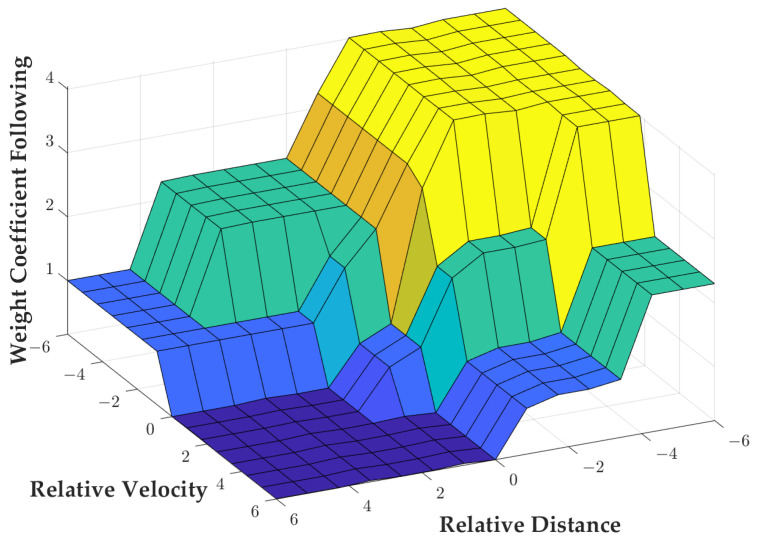
Graph of input and output variables.

Since the output of the fuzzy controller cannot be directly applied to the actual control, the area-centered method is used to solve the fuzzification calculation to obtain the final value of the followability weighting factor *Q* and act on the system. The output variable is also plotted about the input variable function surface as shown in Figure 9.

### 3.3. ACC System Execution Layer Control Method

The execution layer must determine whether the vehicle requires acceleration or deceleration based on the desired acceleration received from the decision layer. It must then convert this desired acceleration into the appropriate actuator commands to achieve the corresponding action. This involves controlling the actuator to apply the required driving torque or braking pressure.

#### 3.3.1. Drive–Brake Switching Logic

In the context of ACC for commercial vehicles, switching between driving and braking states is essential to match the desired acceleration changes. Frequent switching may lead to acceleration oscillation that can affect the actuator, whereas untimely switching can impact the control efficacy of the system. In this research, a rule-based driving–braking switching strategy was implemented. Specifically, the deceleration achieved by the vehicle in a gear-sliding state serves as the threshold value for the driving–braking switching. To simulate the gearbox sliding of the target vehicle, the TruckSim software was used. During the simulation, the throttle opening and braking pressure were set to zero, while the vehicle’s initial speed was set to 120 km/h. The deceleration corresponding to each speed from 0 to 120 km/h was measured and the simulation results were smoothed to derive the driving–braking state switching logic, which is illustrated in Table 5.

#### 3.3.2. Braking Torque Distribution Based on Dynamic Vertical Wheel Load Observation Results

The distribution of vehicle braking force according to the dynamic load of each wheel can make full use of the road friction conditions, thus solving the disadvantage of conservative braking force distribution and ineffective use of road friction that exists in the rule-based braking force-distribution strategy.

When the deceleration demand signal from the ACC system or the brake pedal opening signal from the driver is detected, the braking force-distribution function starts to work. The first step is to calculate the sum of the spring mass of the vehicle, as shown in Equation (32).

(32) $$F_{total}=\sum_{i=1}^{4}F_{zi}$$

The second step is to calculate the braking torque of vehicle demand, as shown in Equation (33).

(33) $$T_{total}=-\frac{r\times a\times \sum_{i=1}^{4}F_{zi}}{g}$$

where *r* is the rolling radius of the wheel.

Finally, the wheel braking force distribution coefficient is calculated, as shown in Equation (34).

(34) $$\lambda_{i}=\frac{F_{zi}}{F_{total}}$$

Therefore, the braking torque of each wheel can be expressed in the form of Equation (35).

(35) $$T_{bi_{}}=\lambda_{i}\times T_{total}$$

#### 3.3.3. Execution Layer PID Control Method

After obtaining the acceleration requirements and braking torque requirements from the decision-making level, the integral-separated PID method is used to control the actuator. The control quantity of the output of the integral-separated PID control can be expressed by Equation (36).

(36) $$u\left(t\right)=k_{p}e\left(t\right)+\beta k_{i}\int e\left(t\right)dt+k_{d}\frac{de(t)}{dt}$$

where β is the switching coefficient of the integral link, $\beta =\left\{\begin{array}{cc} 1 & |e(k)|\leq \varepsilon \\ 0 & |e(k)|>\varepsilon \end{array}\right..$

After setting the deviation threshold ϵ, the deviation between the actual torque and the expected torque is judged; if |e|>ϵ, PD control is adopted to cancel the integral link to avoid excessive overshoot and oscillation due to integral accumulation and if |e|>ϵ, PID control is adopted; integral control is introduced to eliminate steady-state error and improve system control accuracy.

In order to improve the control effect of the ACC system in actual driving scenarios, it is necessary to consider the influence of road surface adhesion changes on the performance of the braking system when designing the control method at the execution layer [28,29]. Therefore, a rule-based anti-lock braking system (ABS) control method based on the execution layer is proposed. In ABS control, the wheel motion state is a combination of rolling motion and sliding motion [30,31]. The rule-based ABS control method uses the wheel slip rate as a reference value and applies corresponding pressure-increase, pressure-holding or pressure-reduction commands to the braking system based on the logical operation result of the actual and preset threshold values of the slip rate. This reduces the proportion of sliding motion in the wheel motion and ensures directional stability and safety during vehicle braking.

**Figure 10:**
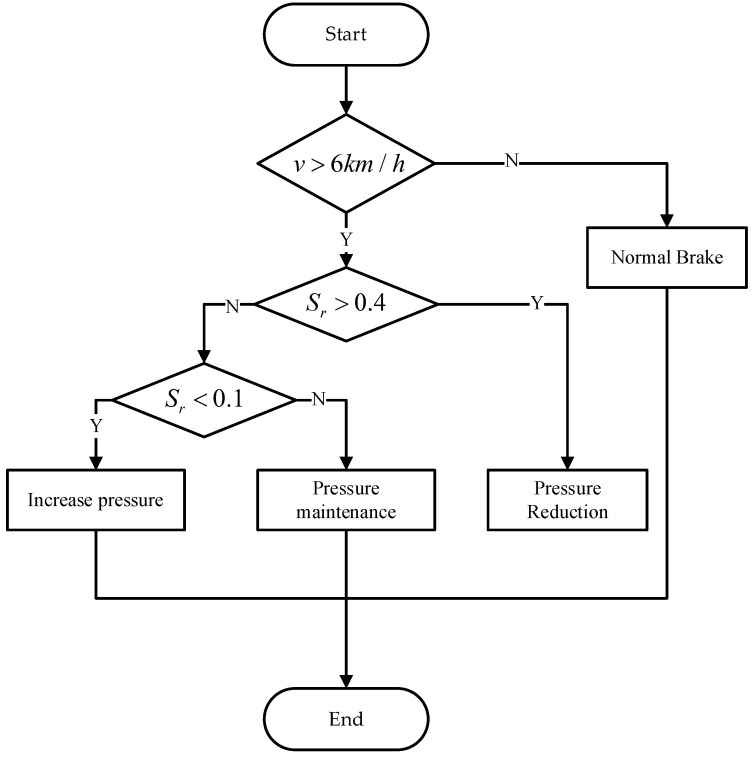
Flow chart of rule-based ABS control method.

The proposed rule-based ABS control logic is shown in Figure 10. After obtaining the vehicle speed and wheel speed signals, the slip rate is calculated according to Equation (12). When braking begins, the ABS function will be triggered when the slip rate is greater than 0.4 and the longitudinal vehicle speed is greater than 6 km/h. The ABS controller sends a pressure reduction signal to the brake actuator. When the slip rate is less than 0.1 and the vehicle speed is greater than 6 km/h, the ABS controller issues a pressure-increase control command. If the slip rate is between 0.1 and 0.4, the braking system pressure remains unchanged from the previous moment. ABS control is exited when the vehicle speed is less than 6 km/h.

## 4. Simulation and Analysis

**Figure 11:**
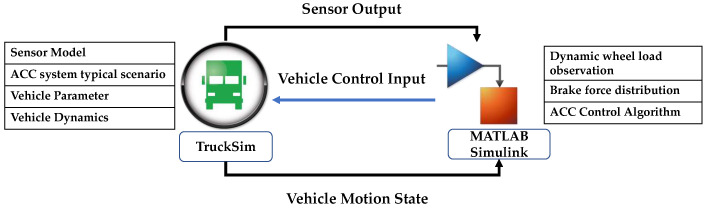
Control strategy simulation platform architecture.

To verify the effectiveness of the proposed control method, a simulation platform was established utilizing Matlab Simulink and TruckSim. The control strategy design for estimating dynamic vertical wheel load was conducted using Matlab Simulink, which acted as the controller, while the TruckSim vehicle model functioned as the controlled object. Additionally, TruckSim was utilized to generate appropriate driving scenarios essential for the functionality of the ACC system. A diagrammatical representation of the co-simulation platform is illustrated in Figure 11. Notably, all of the simulation and model training operations mentioned in this study were performed on a computer equipped with an Intel i7-12700H processor and 16 GB RAM.

### 4.1. Dynamic Vertical Wheel Load Observation and Braking Torque Distribution Results

**Figure 12:**
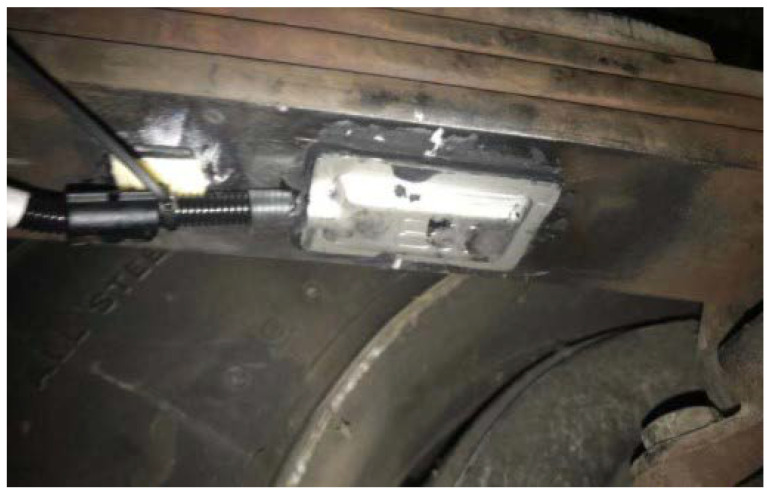
Wheel dynamic vertical load sensor installation diagram.

The dataset used in this paper is derived from the results of real vehicle tests conducted with a cooperative enterprise. Stress–strain sensor strips were installed on the suspension of the test vehicle to measure the dynamic vertical load of the wheels, as shown in Figure 12. The electronic stability control (ESC) system of the test vehicle was equipped with a three-axis accelerometer ADXL313, which can measure the acceleration in the X-Y-Z directions of the vehicle. Signals such as engine speed and torque, vehicle speed and wheel speed were all acquired from the vehicle control unit (VCU) through a CANoe device.

This paper collected data for 20 dynamic parameters that change with time during vehicle operation. The parameter list is shown in Table 6.

After filtering and interpolating the data, a random forest algorithm with 500 decision trees and 3 feature nodes was used for feature selection. Eight data items with high weights were eventually selected for dynamic observation of vehicle wheel vertical load. The selected data items are shown in Table 7.

The total size of the dataset is 11,800 groups. The selected data groups were divided into training set, validation set and test set in a ratio of 7:2:1. The normalized data were trained using FCNN network. The specific parameters and training process of the model are shown in Table 8.

**Figure 13:**
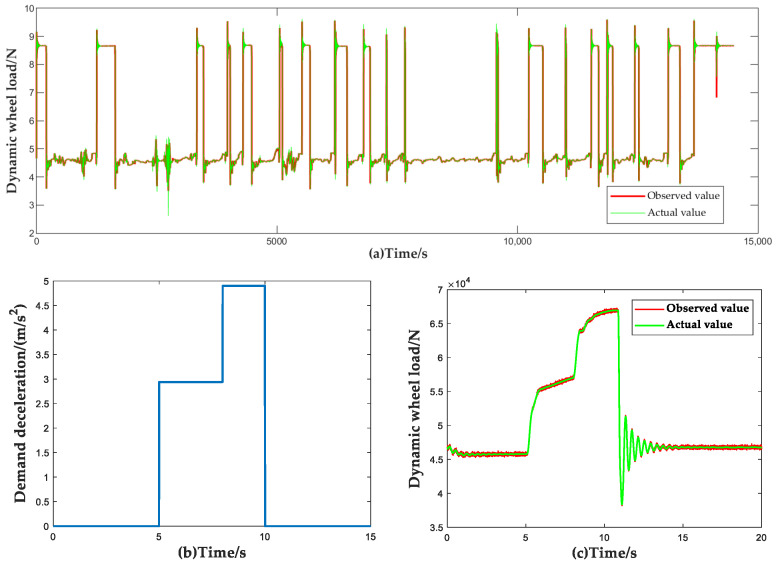
Dynamic vertical wheel load observation results. (Taking the left front wheel of the vehicle as an example.) (**a**) Observations of actual vehicle driving data over a period of time; (**b**) Single braking condition; (**c**) Single brake condition observation result.

To verify the observation accuracy of the vehicle wheel load observer, in the test set, the selected eight types of data were used as inputs to FCNN and the output results were compared and analyzed with the results of the actual vehicle test. Figure 13 and Table 9 show the observed results and estimation accuracy of the dynamic vertical wheel load.

To evaluate the accuracy of the machine learning-based wheel dynamic load observer proposed in this article, a comparative analysis was conducted on the motion parameters of vehicles over a specified period. Figure 13 illustrates the dynamic vertical wheel load observation results. Additionally, Table 9 summarizes the findings obtained from this comparison analysis.

In RMSE and MAE, *n* denotes the sample size, $y_{i}$ denotes the actual observed value and ${\hat{y}}_{i}$ denotes the model predicted value. Smaller values of RMSE and MAE indicate that the predicted value is closer to the true value. The coefficient of determination ($R^{2}$) is used to assess the degree of fit of the regression model and a value closer to 1 indicates a better fit. SSR denotes the sum of squared regressions, SSE denotes the sum of squared errors and SST denotes the sum of total squares.

**Figure 14:**
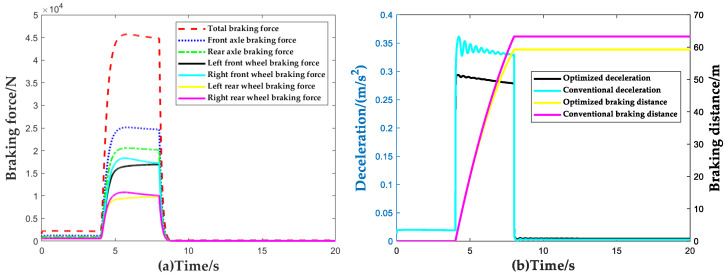
Validation results of brake force distribution strategy. (**a**) Comparison of braking torque distribution; (**b**) Comparison of braking deceleration and braking distance results.

In accordance with the identification results of the dynamic normal wheel load observer, the brake torque of the ACC system is allocated. To verify the control effect of the proposed brake torque allocation method, the results of the brake torque allocation method proposed in this paper are compared with the traditional one based on the fixed proportion of front and rear axles in a single brake scenario ($\beta_{d}=\frac{F_{Bf}}{F_{Br}}=0.55:0.45$, $F_{Bf}$, $F_{Br}$ are the maximum braking force of the vehicle front and rear axle brakes, respectively). The comparison of vehicle deceleration and braking distance under the two methods is shown in Figure 14. It should be noted that, to highlight the performance differences between different brake torque allocation methods more significantly, open-loop control is adopted for deceleration in the comparison process. The simulation scenario is set as follows: the initial speed of the vehicle is 80 km/h and the braking begins at 4 s; the required deceleration is 3.5 m/s${}^{2}$ and the vehicle is released from braking at 8 s.

From Figure 14, it can be seen that the braking force allocation method based on the machine learning dynamic wheel vertical load observer proposed in this paper can effectively improve the vehicle’s braking capability. Compared with the constant proportional braking force allocation method, allocating the braking force based on the vehicle’s dynamic vertical wheel load distribution in open-loop braking scenarios can increase the average deceleration of the vehicle by 18% and shorten the braking distance by 4 m. This significantly improves the utilization rate of road adhesion during the braking process.

### 4.2. ABS Control Simulation Results

To validate the effectiveness of the proposed rule-based ABS control strategy, a simulation verification of the control strategy was carried out on typical road surfaces. The simulation verification scenario was set up as shown in Table 10.

#### 4.2.1. Low Adhesion Road Simulation Result

**Figure 15:**
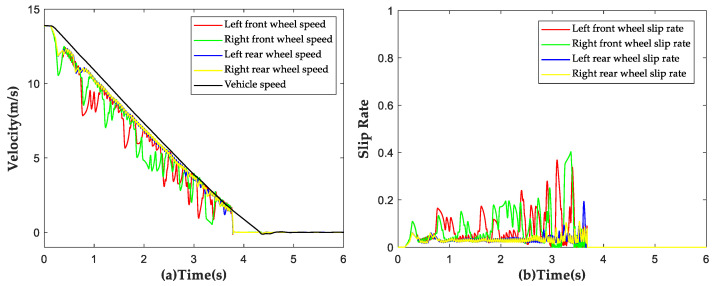
Simulation results of ABS strategy under low adhesion conditions. (**a**) Vehicle speed and wheel speed curves; (**b**) Wheel slip rate curves.

The vehicle was subjected to a braking test with an initial speed of 50 km/h on the aforementioned road surface at μ=0.4, and simulation results are shown in Figure 15. The trends of wheel speed changes under ABS control were found to be consistent with those of vehicle speed changes. No locking of wheels was observed during the entire braking process and ABS disengaged control when the vehicle speed decreased below 6 km/h. The average slip rate of all four wheels was calculated to be 11.23% during the braking process, indicating the effectiveness of the proposed strategy under low-adhesion road conditions.

#### 4.2.2. Split Road Simulation Result

**Figure 16:**
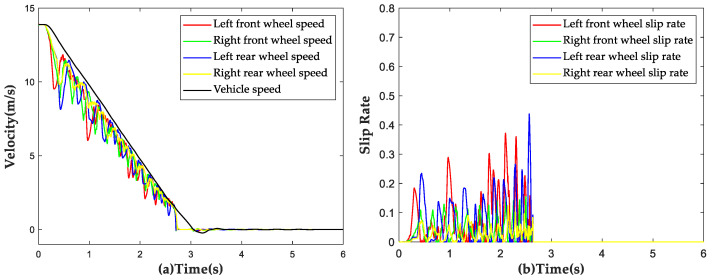
Simulation results of ABS strategy under split road conditions. (**a**) Vehicle speed and wheel speed curves; (**b**) Wheel slip rate curves.

The vehicle was subjected to a braking test with an initial speed of 50 km/h on the aforementioned split μ road surface, with a low adhesion coefficient of μ=0.4 on the left side and a high adhesion coefficient of μ=0.8 on the right side. The simulation results are shown in Figure 16. During the braking process, the average slip rate of the left wheels was found to be 17.43%, with a maximum value of 43.34%, slightly higher than the predetermined threshold value. On the other hand, the average slip rate of the right wheels was 6.14%. No locking of wheels was observed during the entire braking process, demonstrating that the proposed strategy still has a good control effect under the split road conditions.

#### 4.2.3. Bisectional Road Simulation Result

**Figure 17:**
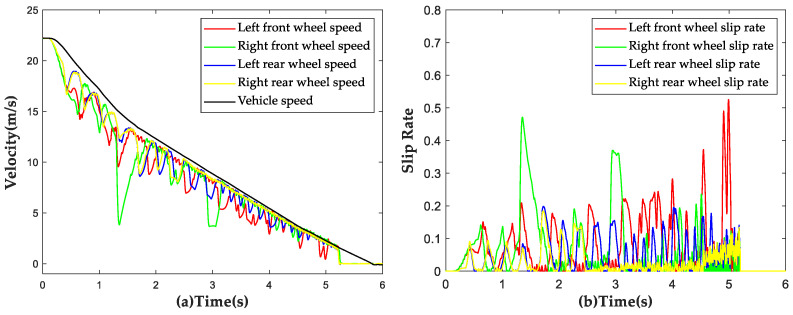
Simulation results of ABS strategy under bisectional road conditions. (**a**) Vehicle speed and wheel speed curves; (**b**) Wheel slip rate curves.

In this section, the road simulation conditions were set to have a road surface adhesion coefficient of μ=0.8 from 0 to 25 m and a road surface adhesion coefficient of μ=0.4 after 25 m. The vehicle was subjected to a braking test with an initial speed of 80 km/h and simulation results are shown in Figure 17. When the vehicle reached the boundary between the two road surfaces, there was a significant increase in the slip rate of the front wheels, but it returned to around 20% within 0.3 s. No locking of wheels was observed during the entire braking process, demonstrating that the proposed strategy can still have a certain control effect under harsh bisectional road conditions.

### 4.3. ACC System Typical Scenario Simulation Result

During vehicle operation, three common scenarios are following a target vehicle, target vehicle insertion and target vehicle braking. These scenarios are utilized to verify the functionality of Adaptive Cruise Control (ACC) systems. The target vehicle scenario is prevalent in high-speed road sections and suburban areas with fewer traffic lights. In this scenario, the ACC system must regulate the vehicle’s speed to smoothly track the target vehicle ahead. The target vehicle insertion scenario requires the ACC system to adjust the vehicle’s longitudinal speed using control strategies to prevent a collision. In the target vehicle braking scenario, the ACC system must promptly brake to avoid collisions. All of these simulation test scenarios compare the Fuzzy Model Predictive Control (MPC) method proposed in this paper with the conventional MPC method that uses fixed weight factors.

#### 4.3.1. Following Target Vehicle

**Figure 18:**
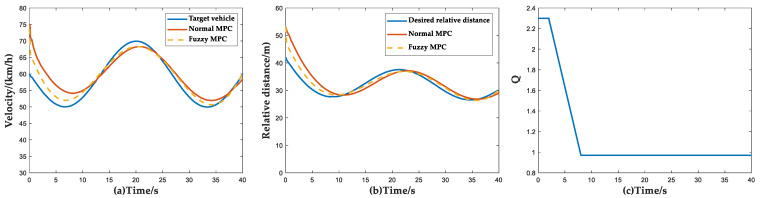
Simulation results of following target vehicle scenario. (**a**) Vehicle velocity simulation results; (**b**) Relative distance simulation results; (**c**) Curves of weight coefficient *Q* versus time and velocity and relative distance.

The initial conditions of the simulation of the following target vehicle scenario are set to the target vehicle driving with variable speed according to the sinusoidal wave-form, the initial velocity of the ACC vehicle is 75 km/h, the initial relative distance is 55 m and the initial velocity of the target vehicle is 60 km/h. The simulation results under this scenario are shown in Figure 18.

In the initial stage of the simulation, the actual relative distance is greater than the desired relative distance, but since the velocity of the host vehicle is higher than the target vehicle, it needs to take braking to adjust the velocity of the host vehicle. Therefore, in the first 2 s at the beginning of the simulation, the ACC system focuses more on the followability index, which is reflected in Figure 18c. After this stage, the weight factor *Q* is stabilized to 1 because the vehicle velocity error and the vehicle distance error gradually converge. According to Figure 18a,b and Table 11, it can be seen that the Fuzzy MPC method has a significant improvement in the control accuracy compared with the normal MPC method.

#### 4.3.2. Target Vehicle Insertion

**Figure 19:**
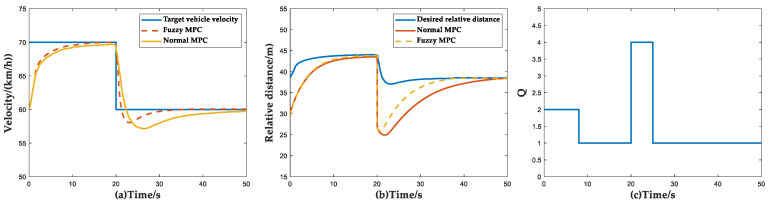
Simulation results of target vehicle insertion scenario. (**a**) Vehicle velocity simulation results; (**b**) Relative distance simulation results; (**c**) Curves of weight coefficient *Q* versus time and velocity and relative distance.

In the simulation of the target vehicle insertion scenario, the initial conditions of the simulation are set as follows: the initial speed of the ACC vehicle is 60 km/h, the original target vehicle is traveling at 70 km/h in front and the initial distance between the two vehicles is 30 m. At 20 s, the vehicle maintains a constant speed of 60 km/h in the next lane, suddenly cuts in and becomes the new target vehicle, at which time the distance between the host vehicle and the target vehicle is 25 m. The simulation results under this condition are shown in Figure 19.

In the first 20 s after the simulation starts, both the Fuzzy MPC algorithm and the normal MPC algorithm can follow the vehicle in front well. At the 20 s mark, the actual relative distance is less than the desired relative distance and the speed of the host vehicle is higher than the target vehicle due to the cut-in of the front vehicle, which creates a risk of collision between the two vehicles. From Figure 19c, it can be seen that the ACC system can accurately determine the priority of following over comfort in this scenario. It can be seen from Figure 19a,b and Table 12 that the Fuzzy MPC method allows the velocity and relative distance to converge to the desired values more quickly. The time required for the velocity and distance to reach the desired values mentioned in Table 12 refers to the time elapsed after the front vehicle intervenes and starts calculating until the velocity and distance converge again.

#### 4.3.3. Target Vehicle Braking

**Figure 20:**
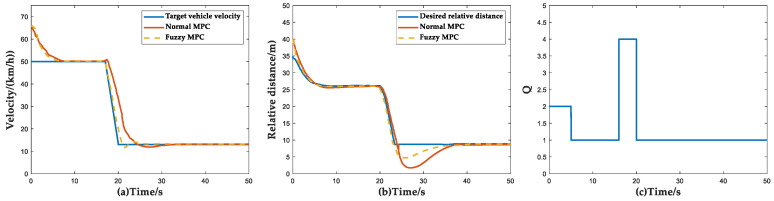
Simulation results of target vehicle braking scenario. (**a**) Vehicle velocity simulation results; (**b**) Relative distance simulation results; (**c**) Curves of weight coefficient *Q* versus time and velocity and relative distance.

In the simulation of this scenario, the initial conditions of simulation for the ACC vehicle and the target vehicle are set as follows: the initial speed of the ACC vehicle is 65 km/h. The target vehicle travels at a constant speed of 50 km/h and brakes suddenly at the 17th second with an acceleration of 3 s and then maintains a constant speed. The simulation results under this condition are shown in Figure 20.

In the beginning, the speed of the host vehicle followed the target vehicle and in the 7th second, the speed of the two vehicles converged and the distance between the two vehicles also converged to the expected value. At 17 s, the target vehicle braked suddenly and the ACC system braked and effectively avoided a rear-end collision, ensuring the safety of the vehicle. From Figure 20a,b, it can be seen that the minimum relative distance of the Fuzzy MPC control method is greater than that of the normal MPC, which guarantees a relative distance greater than $Deltas_{0}$. From Table 13, it can be seen that the following accuracy and convergence speed of the FMPC method are significantly better than those of the conventional control method. Figure 20c shows that the ACC system has higher requirements for safety when the target vehicle brakes, which follows the design expectation of the controller.

## 5. Conclusions

This paper proposes a control strategy for the ACC system of commercial vehicles in actual driving scenarios based on machine learning state observers and Fuzzy MPC, aiming to improve its performance. It consists of a vehicle dynamic normal wheel load observer, a Fuzzy Model Predictive Controller and an integral-separate PID executive layer controller. The design based on the machine learning state observer estimates the dynamic normal wheel load parameters during vehicle travel and applies them to the longitudinal dynamic control of the ACC vehicle, determining the prerequisite for brake force allocation based on the estimation results. This allocation method can increase the braking deceleration by 18% under fixed brake pedal opening. The Fuzzy Model Predictive Controller dynamically adjusts control parameters through fuzzy processing of performance index weight factors to ensure system safety. To verify the proposed strategy, a joint simulation platform was established in the TruckSim-Simulink environment and the system performance was simulated and tested in typical scenarios. The results show that the proposed method improves speed-tracking accuracy (RMSE value) by 41.38% and distance-tracking accuracy by 41.31% in the following target vehicle scenario. In the target vehicle insertion scenario, while improving tracking accuracy, the convergence speed of speed and distance tracking is also increased. It can be proved that compared with previous MPC Adaptive Cruise Control strategies, the ACC system control strategy based on machine learning state observer and Fuzzy MPC can respond to changes in vehicle driving scenarios more quickly and stably. The proposed execution layer control method was verified under simulation conditions on a low-adhesion road, a bisectional road and a split road; the results showed that it can handle changing road conditions well and further improve the safety performance of the ACC system.

Future work will focus on two aspects. First, further research will focus on the robustness of the strategy to parameter uncertainties and external disturbances, especially the applicability of the strategy to different vehicle models and its robustness under more detailed driving scenarios (such as vehicle passing over speed bumps). Second, the proposed control strategy will be validated in real-world vehicle tests.

## Figures and Tables

**Table 1:** Vehicle parameters.

Parameter	Unit	Value
Vehicle mass	kg	11,550
Wheelbase	mm	5500
Front wheel track	mm	2080
Rear wheel track	mm	1870
Wheel rolling radius	mm	530
Frontal area	m${}^{2}$	7.5
Engine maximum power	kW	323

**Table 2:** Brake-related parameter setting.

Parameter	Unit	Value
$A_{b}$	m${}^{2}$	0.005
$\eta_{b}$	-	0.99
$\mu_{b}$	-	0.25
$r_{b}$	m	0.3
$c_{b}$	-	3

**Table 3:** ACC System Constraints.

Constraint Name	Constraint Expressions
Safety	$\Delta s\left(k\right)\geq \Delta s_{0}$
Following	$\delta \left(k\right)\to 0,v_{rel}\left(k\right)\to 0,when(k\to \infty$)
Comfortability	$j_{C\min}\leq j_{C}\left(k\right)\leq j_{C\max}$
Velocity restraints	$v_{\min}\leq v\left(k\right)\leq v_{\max}$
Control variable constraint	$u_{\min}\leq u\left(k\right)\leq u_{\max}$

**Table 4:** Fuzzy control rules table.

$\delta \left(k\right)/v_{\mathrm{rel}}\left(k\right)$	NB	NS	ZO	PS	PB
NB	PB	PB	PB	PB	PM
NS	PB	PB	PB	PM	PS
ZO	PM	PM	PS	PS	ZO
PS	PM	PS	ZO	ZO	ZO
PB	PS	PS	ZO	ZO	ZO

**Table 5:** Driving–braking switching logic.

Switching Logic	Vehicle State
$a_{des}\geq a+\Delta h$	Driving
$a_{des}\leq a-\Delta h$	Braking
$a-\Delta h<a_{des}<a+\Delta h$	Keep the original state

**Table 6:** Original data classification.

Number	Parameter Name	Number of Parameters
1	Engine speed and torque	2
2	Front wheel turning angle	1
3	Transmission ratio	1
4	Wheel brake pressure	4
5	Wheel speed	4
6	Wheel slip rate	4
7	Vehicle longitudinal acceleration	1
8	Sprung mass vertical velocity	1
9	Yaw rate	1
10	Longitudinal vehicle speed	1

**Table 7:** Selected data items.

Number	Parameter Name	Number of Parameters
1	Engine speed and torque	2
2	Wheel brake pressure	4
3	Vehicle longitudinal acceleration	1
4	Sprung mass vertical velocity	1

**Table 8:** Detailed Parameter Setting.

Parameter Name	Value	Parameter Name	Value
Number of hidden layers	2	Traning epoch	1000
Number of hidden layer nodes	128/64	Training set size	8260
Learning rate	1 × 10${}^{-4}$	Validation set size	2360
Batch size	128	Testing set size	1180
Optimizer	ADAM	Weight decay	1 × 10${}^{-4}$

**Table 9:** Accuracy analysis of observation results.

Evaluation Criteria	Calculation Formula	Calculation Results
RMSE	$RMSE=\sqrt{\frac{1}{n}\sum_{i=1}^{n}{\left(y_{i}-{\hat{y}}_{i}\right)}^{2}}$	320.04/*N*
MAE	$MAE=\frac{1}{n}\sum_{i=1}^{n}|y_{i}-{\hat{y}}_{i}|$	107.41/*N*
$R^{2}$	$R^{2}=\frac{SSR}{SST}=1-\frac{SSE}{SST}$	0.9997

**Table 10:** ABS simulation conditions.

Road Setting	Initial Speed
Low adhesion road	50 km/h
Split road	50 km/h
Bisectional road	80 km/h

**Table 11:** Comparison of the control accuracy of different methods of following target vehicle scenario.

	Normal MPC	Fuzzy MPC	Performance Enhancement
Velocity RMSE	3.80 km/h	2.23 km/h	41.38%
Relative distance RMSE	3.72 m	2.18 m	41.31%

**Table 12:** Comparison of the control accuracy of different methods of target vehicle insertion scenario.

	Normal MPC	Fuzzy MPC	Performance Enhancement
Velocity RMSE	6.82 km/h	6.04 km/h	11.38%
Relative distance RMSE	6.37 m	5.98 m	6.31%
Time required for velocity to reach reference value	25.55 m	12.16 m	13.39 s
Time required for distance to reach reference value	24.83 m	16.25 m	8.58 s

**Table 13:** Comparison of the control accuracy of different methods of target vehicle braking scenario.

	Normal MPC	Fuzzy MPC	Performance Enhancement
Velocity RMSE	6.36 km/h	5.69 km/h	10.57%
Relative distance RMSE	4.82 m	3.20 m	33.62%
Time required for velocity to reach reference value	9.07 m	5.23 m	3.84 s
Time required for distance to reach reference value	14.64 m	12.11 m	2.53 s

## Data Availability

Not applicable.
